# Stress, psychosocial resources and possible interventions: a qualitative study among dental students

**DOI:** 10.1186/s12909-024-06472-1

**Published:** 2024-12-18

**Authors:** Lisa Guthardt, Clara Niedworok, Thomas Muth, Adrian Loerbroks

**Affiliations:** Institute of Occupational, Social and Environmental Medicine, Centre for Health and Society, Heinrich Heine University and University Hospital Düsseldorf, Moorenstr. 5, Düsseldorf, 40225 Germany

**Keywords:** Dental students, Psychological stress, Mental health, Qualitative research, Health resources, Focus groups, Dental education

## Abstract

**Background:**

Prior studies found that dental students are affected by various stressors during their studies. Those stressors can exert adverse effects on their (mental) health. Our study addresses the lack of qualitative data on students’ perspectives by exploring perceived stressors and resources among dental students and interventions suggested by them. The results of our study can contribute to the development of better preventive measures and interventions.

**Methods:**

In total, 57 dental students enrolled at a dental school in Germany participated in seven focus groups in the summer semester 2019 (May to July). Discussions were facilitated using a topic guide, and data collection was conducted until thematic saturation. All discussions were audio-recorded, transcribed and content-analyzed using MAXQDA.

**Results:**

Key emerging stressors were related to the organization of the study program, a lack of digitalization, practical tasks, the examination system, the work/study environment and social interactions. Resources encompassed, e.g., good organization, practical courses, patient work and valued feedback. Interventions suggested by students included regular meetings to enhance collaboration, improved communication between staff and students, a central coordination unit, fixed evaluation criteria and the integration of physical exercises and physiotherapy in the study program to prevent neck and back pain.

**Conclusions:**

Known stressors for dental students and new aspects (e.g., concurring exams or obligatory brands) emerged from our data. Interventions suggested by the students included the use of digital learning platforms, communication training or the improvement of evaluation processes. Additional research, e.g., to explore perspectives of teaching staff and other stakeholders is necessary to gain more insights into study conditions and ways to reduce stress among dental students.

**Supplementary Information:**

The online version contains supplementary material available at 10.1186/s12909-024-06472-1.

## Background

Students in general are especially vulnerable to psychological distress [1]. Due to high demands, dental studies are considered particularly stressful and one of the most challenging subjects of study [2], and dental students are exposed to many different stressors [3]. At the same time, perceptions of their future profession among dental students are very positive (e.g., with regard to social status or a positive connection to technology, i.e., dentistry as a profession that has a direct relation to technology) [4]. In a systematic review of stress in dental students, academic stressors (examination and grades as well as the general workload), faculty-related stressors (school-specific regulations and rules, receiving criticism and inconsistent feedback from teaching staff) and clinical aspects (dealing with difficult patients and having difficulties in learning clinical procedures) were listed as the main sources for stress in students [5]. Other stressors that have already been identified include schedule pressure, patient care responsibility [3], fear of failing a course/year, lack of time to complete assignments, differences between expectation and reality of dental school, patients who are late or cancel their appointments, a lack of cooperation by patients and difficulties finding suitable patients, financial responsibilities or a lack of recovery time [1, 6, 7].

Academic stress can exert adverse effects on students’ physical and mental health and can increase the risk of depression, anxiety, burnout or even suicidal ideation [8–10]. Stressors can also contribute to, e.g., physical illness, antisocial behavior or poor academic performance [11]. In a survey with 163 students of dental medicine in Germany, it was found that the perceived quality of life decreases in the course of the study and that symptoms of depression increase with every semester [12]. Stressors are thus known to negatively impact the health of students. The development and implementation of interventions aiming at reducing or preventing stressors is therefore necessary in order to primarily improve students’ mental well-being and to also enhance their performance during their studies and later in their profession.

A systematic review found that only 9.1% of studies related to stress and stress levels among dental students were of qualitative nature [5]. Data concerning interventions to improve well-being in the dental sector is even more scare and findings need to be expanded [13]. Yet, qualitative approaches targeted at dental students are especially important as qualitative approaches generally allow for the collection of in-depth insights and real-world experiences of individuals [14]. These insights are useful in order to improve study conditions in the long run. In one qualitative study from Denmark, 12 final-year dental students were questioned on their experiences with a mentoring program that had been implemented at the university [15]. However, the study’s focus was on early clinical experience, individual reflection, social perspectives and thoughts of dropping out of dentistry, and all questions were related to the specific mentoring program [15]. Additionally, only female students were interviewed, which may limit the range of potential views that could be explored, and the number of interviews conducted might not have led to thematic saturation, i.e., to the point where it seems unlikely that new findings emerged from the material, which can be seen as a quality criterion of qualitative research [16]. A Canadian mixed-methods study dealt with sources of stress in dental students, but only six qualitative interviews were conducted, which might not cover all views (i.e., again, it remains elusive to what extent thematic saturation was reached) [17]. In another study, data on dental students’ perceptions of the strengths and weaknesses of the dental school curriculum in North America were collected on a larger scale, but data acquisition was done in an open-ended written format, no discussions or focus groups were conducted, there was no personal interaction, and the survey might therefore lack in-depth insights [18]. There are some qualitative studies concerning perceived stress among dental students with 24, respectively 37 participants [19, 20], but both studies focused on perceived stressors in the students’ transition from the pre-clinical to the clinical section. Thus, these two studies do not cover the complete study time, and the students’ suggestions on how to reduce stress are also targeted at the transition phase instead of providing general starting points for interventions [19]. There are also several studies that addressed specific perceived effects of certain interventions from the students’ perspective (e.g., yoga sessions [21], an educational concept for risk-oriented prevention [22], the incorporation of self-care strategies in the curriculum [23], mind-body wellness training [24] or cognitive reappraisal technique to reduce stress [25]). Comprehensive studies including stressors and suggested interventions by students also exist for medical studies (e.g [26–28]). A recent mixed methods study provided further comprehensive insights into dental and endodontic-related stress in undergraduate students, revealing stressful domains in the study (performance pressure, workload and clinical training) and also providing insights into suggestions for improvement on the part of the students (e.g., introducing a patient management team, including more laboratory sessions in the curriculum in order to practice more, more support with administrative work or first-year and second-year students assisting students in higher semesters to provide more assistance for them and teach students from first semesters practical knowledge early on) [7].

To the best of our knowledge, what is still missing in the research literature is more comprehensive qualitative research on stressors, resources and potential interventions among dental students instead of focusing on individual aspects like the transition from the pre-clinical to clinical section or the effects of mentoring programs. More qualitative research collecting data until it is plausible that thematic saturation is reached is also needed. Instead of merely assessing stressors and possible consequences of stress (as done before in several studies), more engagement with resources and suggested solutions is necessary in order to not only reduce or avoid stressors, but to identify approaches to strengthen resources and develop adequate prevention measures for students. With our qualitative study, we seek to expand previous findings on stressors, resources as well as possible interventions in dental education from the perspective of dental students. We want to address the current research gap by providing insights from focus groups, which are particularly suitable to devise interventions: By talking to dental students, we are likely to collect measures or interventions that the students themselves assess as acceptable and effective. Additionally, focus group discussions are useful because participants can be more inclined to think and talk about aspects of their daily study life [29].

As study conditions for dental students need to be improved further and due to the current lack of research in this matter, the goal of our study is to provide comprehensive insights into perceived stressors, resources and preferences for possible interventions from the students’ perspective. We pursue the following research questions:

i. Which stressors do students in dental studies perceive at the site?
ii. Which individual resources are considered as beneficial by students?
iii. Which possible interventions do students suggest in order to avoid or reduce stress?

## Methods

### Research team

Several researchers were involved in the conduction of the study. TM is a psychologist by training and teaches occupational medicine at the university of Düsseldorf, conducts research on work stress and student health and is experienced in qualitative research, including the facilitation of focus groups [28, 30]. AL has a background in health sciences, is an experienced qualitative researcher and does research on work stress and health, including students’ mental health and stress [31, 32]. LG is a researcher, who is experienced in qualitative methods and who has a degree in English studies and in literary translation [33, 34]. At the time of data collection and analysis, CN was a dental student in Düsseldorf herself.

### Study participants

A convenience sampling method was used for this study: CN approached study participants on the university campus and in the clinic. She talked about the planned research project in dental seminars and main lectures and encouraged students to participate. Those who were interested were added to a list in order to obtain further information on where and when the group discussions were to take place. The two selection criteria were current enrolment in the dental study program at the university and willingness to participate in focus group discussions. Additional selection criteria were not applied. Cinema tickets were offered as an incentive. Overall, we were able to include 57 out of approximately 300 enrolled dental students. Concerning age, 74% of the participants were between 20 and 30 years old, whereas the youngest one was 18 and the oldest was 35 years old. The majority of participants were female (*n* = 84%), which is slightly more compared to the study program in general. The dental study program at the site encompasses 5 study years with 2 semesters each, i.e., a total of 10 semester followed by the final state examination. We were able to include students from the second, fourth, sixth, seventh, eight and tenth semester. As data collection took part in the summer semester and students start the study program in the winter semester only, no students from the first, third and fifth semester could be included. In the clinical section (from the sixth semester onwards) course admissions take part six-monthly. No students from the ninth and eleventh semester or above participated, potentially due to the smaller size of the totality of students in the ninth semester and the state examination following the tenth semester (i.e., students might have been too busy with exam preparation to take part in the study).

### Data collection

Participants were recruited in the summer term 2019 among dental students enrolled at the dental school at a German university. In total, 7 semi-structured focus group discussions with 57 participants from the preclinical (*n* = 3) and clinical (*n* = 4) section were conducted in the summer term 2019 from May to July. Each focus group consisted of five to thirteen students of the same section to ensure they experience similar contexts and are able to share their views with the group. Discussions were facilitated by TM, who is not involved in teaching dental students. At the beginning of each group discussion, TM briefly presented the goal of the study, the procedure and further information in advance (e.g., data protection). He then posed some introductory questions related to positive and negative aspects of the studies: Participants should talk about how they felt and how they have experienced their study life so far. Additional key questions (concerning stressors, resources and suggestions for improvement) were brought up by the facilitator whenever it seemed appropriate with regard to the research questions. More detailed information about the structure and the questions asked can be found in the topic guide (see Additional Material 1). All students were encouraged to take part in the discussion. Numbers instead of names were used to address participants so that all students were motivated to speak freely and without concerns with regard to the audio recording. Group discussions took between 59 and 89 min (mean = 74 min). Ethical approval was obtained by the ethics committee of the medical faculty of the University of Düsseldorf before data collection (Ethics Committee Düsseldorf, Studie 4041, approved on the 23.03.2015). Face-to-face group discussions were carried out until data saturation seemed likely, which means that no new findings occurred and can be seen as a quality criterion of qualitative research [16]. All group discussions were recorded, digitally transcribed by an external company and then analyzed via qualitative content analysis according to Mayring and Kuckartz [35, 36]. Additionally, CN took notes during the focus group sessions.

### Data analysis

The software MAXQDA 2020 (VERBI GmbH, Berlin, Germany) was used for the analysis of the data. First, individual statements were summarized and paraphrased by CN to gain a broad overview of the material. Key statements of all seven focus groups were written down in the form of memos. In the next step, all interviews were initially coded by CN according to a category system that was created both deductively through relevant questions and issues that occurred in the interview guideline and inductively based on the material. The category system was tailored to the material in order to cover all relevant aspects. After two coding rounds, the results were presented to AL, and the category system was extensively discussed and adapted, which led to a third coding round. After this, the category system was checked and revised by LG. The material was once again completely coded by LG, and additional categories were formed whenever it seemed necessary. It was also discussed whether the determined categories were appropriate and sufficiently distinct, i.e., whether certain categories lacked differentiation, were too similar or not clearly determined. All findings were discussed with CN. The results were presented to AL and TM, and a joint reflection session led to the preliminary version of the category system. Finally, LG re-coded the material once again because a more fine-grained analysis was deemed necessary. Due to her background and language skills (degrees in English studies and literary translation), an in-depth analysis was possible, which led to the final version of the category system.

## Results

Table 1 displays stressors perceived by the students as well as corresponding interventions suggested by them. In Table 2, resources mentioned by the participants are summarized, sorted by main codes. All quotes from the data material were translated into English and can be found in the text as well as in Additional Material 2 and 3 (additional quotes, references A-P for stressors and A-L for interventions).

**Table 1:** Overview of stressors perceived by the dental students and corresponding suggestions for improvement

Main Codes	Specific stressors (summary)	Improvements suggested by the students
Organization of the study program	• poor organization of the study program (e.g., decisions concerning course organization were made by people not involved in teaching and who were perceived to be unfamiliar with course-related conditions) • lack of a central coordination unit/lack of contact persons • lack of availability of staff and support (e.g., insufficient time to ask questions, not enough supervision during practical exercises) • lack of cooperation and a negative atmosphere between the clinical departments • information often not provided in time or changed frequently, lack of general information concerning the study program (e.g., work intensity, expected costs) • great workload and lack of free time • unnecessary waiting times • lack of course places/no guarantee of receiving a place as well as long waiting times in case you did not get a place	• implementing integrated seminars; semester schedule should be planned together with dental and medical students • having a person who functions as a central coordinator; more contact persons students could address when having problems mandatory rule: Ambulance service should only be done by doctors who do not supervise students at that time • more communication between departments and persons; weekly or monthly exchange meetings, also to plan course and exam dates; personal problems should not be discussed in front of students (more professional behavior) • timely information on plans and courses at the beginning of each semester; receiving a complete overview over the study program (i.e., which courses to take in which semester); notifications about what is most important in the upcoming semester • study time should be available/regarded, one week time to only learn, more time to deal with topics in-depths • providing enough course places for all students; accepting fewer people • hiring an external quality manager
Digitalization	• perceived lack of digitalization • learning material often not available in digital form • delays in receiving certificates for passed courses/exams • patient files are not digitally available	• more effective use of the available course-management platform (message function, upload of lectures, course (de-)registration) • providing exam results, certificates and lectures digitally • complete digitalization and availability of all clinical documents, files and patient information (appointments etc.) • more effective use of the university mail; mailing lists to provide information
Study content/ practical tasks	• challenges in practical tasks • recruitment and treatment of patients • lack of time when treating patients • contacting patients (e.g., to confirm or postpone an appointment) • high responsibility when treating patients • missing autonomy and scope of action in the treatment of patients (i.e., wish to experience professional growth)	• possibility to access preparation rooms in the evening for those who still want to practice • filming a short clip to promote the dental clinic
Examination system/ evaluation criteria	• lack of impact on their grades • lack of objectivity • diligence and performance sometimes do not seem to matter • no mistakes allowed • number, content and scheduling of exams • difficult relation to their examiners as they were often their lecturers/supervisors as well • feedback and evaluation sheets (missing anonymity, not possible to give honest feedback without consequences) • arbitrariness concerning exams and in the study program in general (lack of reliable evaluation criteria) • course failure due to missing patients	• also grading posture in technical tasks on body models (i.e., patient would not be harmed in the process, adequate sitting posture) • leaving more room for mistakes • in case of failed exams: feedback talks to find out what needs to be improved; possibility to repeat an exam/a course within the semester; improvement of exams, new exam questions in every exam, more focus on topics that are important for dentists (e.g., dental anatomy instead of every part of the brain), more time to deal with topics and to effectively learn them (instead of learning a great amount for an exam and forgetting all of it right away) • always different order in published lists with results/grades (for more anonymity); taking students’ feedback seriously, valuing students’ perspective, improving aspects that have been criticized, using feedback/evaluation as an option to improve courses, not only evaluating teaching and courses but also the students’ health/condition, asking how students feel/are (to prevent the feeling that nobody cares about this) • fixed, general evaluation criteria in every semester, independent of individuals (subjective evaluation only for minor aspects like fine tuning); clearly mentioning the evaluation criteria at the beginning of each course (with examples/being shown how to do it) • quality over quantity, missing patients should not lead to failure • no compulsory attendance anymore (to allow for individual setting of priorities/which courses are most important at the moment), more focus on technical work instead of signatures and attendance, more understanding and options in case of illnesses
Study/work environment	• broken/missing material, or material not up-to-date • necessity of sharing with too many others • costs for their studies (financial burden, missing support from the university) • obligation to buy certain brands • material lists inaccurate or not up-to-date (i.e., they often seemed to buy more than they in fact needed) • missing financial support from the university • inappropriate workplaces (e.g., too small rooms, no lockers) • somatic stressors (bad posture) • physical/chemical stressors (fine dust, plastic smells)	• exchange between staff concerning sets and material to see if certain expenses could be saved (e.g., in case of almost similar material) • recommendations instead of obligation • student body/university could purchase instruments and material, loan or fees, insurances • retreat or break room for students, two-part lockers for clothes • implementing physiotherapy, being shown how to sit, how to save/preserve the back, wearing corsets, learning some exercises for the back to prevent back pain and tensions
Social interactions	With (teaching) staff • unfriendly or harsh treatment • lack of appreciation, understanding and respect • indifference of teaching staff With fellow students • sometimes tense atmosphere among students • sometimes less mutual support because of competitive behavior • perceived hierarchies (between students from different semesters, i.e., students from higher semesters are prioritized)	• also being addressed formally when addressing someone else formally • being shown how to do things properly • establishing more guidance and support • teaching staff should participate in courses on how to teach efficiently and appropriately • reinforcement of the student body
Personal stressors	• bad conscience • lack of time for family, friends and recovery • difficulties for students from other countries • financial stressors • irritated work-life-balance • neglecting own health • time and performance pressure (e.g., constant stress and fear of not being good enough, pressure to finish in the standard period of study)	• priority for students from other countries for courses in the semester • promoting sports and the importance of sports

**Table 2:** Overview of resources perceived by the students, sorted by main codes

Main codes	Specific resources (summary)
Organization of the study program	• good organization of classes • adequate student-supervisor ratio (enough supervisors for the students) • timely information on scheduling and courses • enough time to complete assignments
Digitalization	• noticeable efforts of teaching staff to improve digitalization and implement new methods or technologies (e.g., high-tech scanner, 3D printing) • implementation of technological features for an improved patient care
Study content/ practical tasks	• certain study and course contents • being able to make own decisions • gained qualifications and experiences • practical courses and work with patients • personal progress
Examination system/evaluation criteria	• having enough time for tasks and submissions • feedback/criticism that matters • the positive feeling of having an impact on the organization of the study program • familiarity in the study program (connection to supervisors/examiners)
Study/work environment	• organized and clean workspace • functioning workplaces • availability of material • usefulness of certain equipment and materials that students had to buy themselves (e.g., dental loupes) • modern and new buildings
Social interactions	With patients • positive experiences with patients With (teaching) staff • positive experiences with teaching staff • teaching staff students could approach in case of questions or problems • being taken seriously and feeling valued With fellow students • mutual understanding • support from fellow students • course/semester speaker • friendships among students • being able to rely on each other • strong team spirit and solidarity
Individual and other resources	• fun • future prospects of becoming dentists • having a part-time job • doing sports • travelling • support from partner, family and friends • strong will and resilience • financial support

## Stressors

### Organization of the study program

Several students described that the lack of a central coordinator seemed to lead to organizational problems and additional burden due to missing responsible contact persons [A; B]. Mainly participants from the clinical section (students in the sixth semester and above) highlighted the perceived lack of cooperation and tense atmosphere between the clinical departments (e.g., radiology, prosthetics). The departments did not seem to make arrangements or consult each other concerning course or exam planning and patient care (e.g., exams or courses taking place at the same time).

“Two departments schedule an event at the same time, and they tell you: ‘You must be present’. And none of them is willing to reschedule it. Sometimes, they even schedule two exams at the same time. Then we students have to chase and beg them, so that they might take pity on us and reschedule one of the events.”

Students also perceived gossiping, internal dispute and resentment between staff from different departments, which was deemed unprofessional [C].

### Digitalization

Mainly participants from the clinical section described a general perceived lack of digitalization. They said that necessary learning material (e.g., presentations, records of lectures) was often not accessible in digital form.

"In all departments you ask your lecturers: ‘Could you upload the lecture on ILIAS?’ And they tell you something like: ‘I don’t know how to do that.’ And I just wonder why, because they’re working at a university, and I think they should know better. Or they simply tell us: ‘No, we don’t do that’."

Students also explained that patient files only existed in a physical form, which often led to additional work and stress (e.g., files being lost or had to be searched) [D].

### Study content/practical tasks

Students described feeling overwhelmed by practical tasks or techniques especially when doing them first-time or without sufficient guidance and practice [E]. Some students perceived stress related to the recruitment and treatment of patients (finding appropriate patients for practical tasks). They also explained that this could lead to a stressed relation to patients and a lack of time during treatment (e.g., not enough time to talk to patients).

“You see this person as a service and not as a human being. I always think, okay, I don’t have enough time. I can’t start a conversation with this person right now. I can’t ask them something about their family, which is something that I actually like to do, because I don’t have time and I have to work very quickly. Maybe there will be some time for it at the end. But during the treatment I can’t ask any personal questions, because I don’t have enough time.”

Concerning patient interaction students felt left alone in some cases (i.e., no adequate preparation to communicate with and treat anxious patients or perceived lack of basic psychological knowledge of social interaction) [F]. Stressors related to the interaction with patients were exclusively mentioned by students in the clinical section, since patient treatment does not start before this section at this dental school.

### Examination system/evaluation criteria

Participants described that the passing and grading of a course often seemed to be determined by external factors (e.g., the availability of patients or the mood of examiners) and talked about a perceived lack of reliable evaluation criteria [G; H].

Students also explained that in order not to be perceived negatively and to avoid standing out, they were often reserved and did not dare to ask questions or criticize staff.

"As soon as you get negative attention, you are labeled and you’re lost. This place is quite small. There’s a lot of talking, lots of rumors, and you can’t easily get rid of them. It’s the same when you stand out in a positive way, they might eventually tell you that you’re not so good anymore suddenly."

Students mentioned that teaching staff often did not seem to accept criticism or take it seriously (i.e., laughing, getting upset in front of the students after receiving negative feedback) [I]. The aspect of missing anonymity in teaching evaluations was also mentioned by some participants. Due to the generally small number of students in the study program, it seemed to be possible to recognize handwriting, or evaluation sheets had to be handed in together with a test or exam [J; K].

According to the students, these factors led to reluctance on their part, i.e., they did not want to give honest feedback anymore and were frustrated because evaluations often seemed to be useless.

### Work/study environment

According to the students, material was often either completely missing, broken or had to be shared with too many people (e.g., not enough disposables such as gloves) [L].

Some participants felt that large sums of money (i.e., up to 6.000€ in the first semester; 12.000€ in total) seemed to be necessary to buy predetermined equipment (e.g., specific dental suitcases) and to successfully finish the studies. For the students, this created much pressure (e.g., because failing a class or waiting a semester to take an exam again always meant additional costs for them) and was perceived as a financial burden [M]. They also perceived the need to buy specific brands in order to pass classes, which was described as problematic.

"There was a huge list of these companies. Some of us bought used cases containing tweezers from other companies. And then they said no, you have to get it from the specific company, but in the end it didn’t matter."

Further aspects perceived by the students involved physical (e.g., bad posture when treating patients) as well as environmental stressors (e.g., strong plastic smells, fine dust pollution).

"It’s a pity […] we are not shown how to sit properly. We should learn how to do this at the beginning. […] What is good for our health? Because you often sit so crookedly and bend yourself. And you haven’t adjusted the chair height, you haven’t adjusted the patient. And nobody pays attention to that. They only tell you to do your work and do it well. But they don’t show us how to do it best and how to sit properly. Then you get back pain, you’re having tensions and so on."

### Social interactions

Fellow students were sometimes perceived as stressors by the participants. They explained that due to the existing pressure and limited course places, the atmosphere between fellow students sometimes seemed to be uncooperative and competitive (e.g., less mutual support, selfishness).

“A lot of people become very selfish. And that doesn’t lead to a good atmosphere among the students. Some students don’t really help out others, since they know that if others fail, they might be more likely to get a place. As a result, many students only focus on themselves. And I think some of them are also happy when others fail. So, in any case, it’s not really cooperative.”

Finally, students expressed that they often felt harshly or unfriendly treated by teaching staff. This was especially relevant in the clinical section, because this is the part of the study program where dental knowledge is conveyed in courses and lectures. Students seemed to perceive a lack of appreciation, understanding and respect and did not have the impression that teaching staff really cared about them and their success [N]. For the students, this led to dissatisfaction, disappointment and a feeling of being left alone with other stressors [O].

### Personal stressors

Some students mentioned having a guilty conscience (e.g., when engaging into free time activities instead of learning) or did not seem to have any time at all for other activities, friends and family. This also seemed to prevent them from adequate recovery.

Difficulties for students from other countries were also expressed (e.g., not being able to visit family in the semester break). Other aspects perceived were the inability to relax, financial stressors (e.g., debts, the need for a part-time job), a poor work-life-balance (e.g., lack of free time, missing vacation) or the neglect of their health (e.g., going to university when being ill, no time for sports) [P].

## Resources

Participants mentioned different resources, an overview of which is displayed in Table 2. For reasons of limited space, these resources cannot be described in more detail in the context of the main text, but they cover aspects such as noticeable efforts of teaching staff to improve digitalization and implement new methods or technologies, work with patients, the future prospect of becoming a dentist and general resources such as mutual understanding among fellow students, financial support, support from family, partner and friends as well as resilience and fun. A more detailed text version can be found in Additional Material 4.

## Potential interventions

### Organization of the study program

To enhance collaboration between the different departments, participants suggested more communication and exchange between them (e.g., weekly or monthly meetings to discuss seminar/semester plans, courses and exams).

“They should all get together once a week and talk about it: Let’s see. This is our course. This is your course. How can we organize the courses so that everything works out for the students?”

Some students wished for timely information on plans and courses at the beginning of each semester and an overview over the study program (i.e., which courses/exams should be taken when). Finally, a central coordination unit was desired because students were missing contact persons they could address with problems [A].

### Digitalization

Several participants wished for a complete digitalization of all clinical documents (e.g., patient files, therapy plans, appointment schedules) so that everyone would always be up-to-date and could have access to all information [B]. It was also favored to provide exam results, certificates and lectures digitally and to use the university mail to distribute information more often [C].

Finally, students proposed that the course-management platform used by the university should be used more frequently (e.g., for messages, course (de-)registration and to upload lectures).

“The university has so many opportunities to do this […] digitally. We have [an online platform], where you can upload lectures, send messages, register for or withdraw from courses. There are so many possibilities, and they are simply not used.”

### Study content/practical tasks

To increase the students’ scope of action, some participants wished for the possibility to access preparation rooms in the evenings (for those who wanted to practice more) [D]. It was also suggested that filming a short clip to advertise the dental clinic and options for patients could be useful to recruit more patients and to spread information [E].

### Examination system/evaluation criteria

Some students proposed feedback talks after a failed exam in order to find out what could be improved [F]. Evaluation criteria played an important role as well. Students wished for fixed, general evaluation criteria in every semester in order to avoid perceived subjective and arbitrary assessment by teaching staff [G].

Some students suggested that the evaluation could be expanded: Instead of evaluating teaching and courses only, the students’ health/condition could also be inquired (e.g., asking students how they feel) to counteract the feeling that nobody cares about this.

“I think it would be a good idea to not only evaluate the teaching, but perhaps also how the students are feeling. So basically, similar to what we’re doing at the moment, where you simply ask, […] how the students are actually doing. Because I don’t think that the lecturers are interested in that at all. Just to find out how things are going, if we are doing well.”

### Work/study environment

It was suggested that departments and teaching staff could exchange information on material lists to see if certain expenses could be saved (e.g., in case of almost similar material for different courses) or that the student body could purchase instruments and material to lend to the students. Some students also explained that re-selling material would be a good option to save some money or that the university should provide more material (e.g., material that could be borrowed by the students for a fee) [H].

An insurance to prevent students from paying additional costs in case of lost or broken material was also suggested. More health prevention regarding back pain was also viewed as useful (e.g., physiotherapy blocks, more focus on how to sit properly, learning exercises to prevent back pain and muscle tension).

“It could also be a bit fun, that maybe someone from physiotherapy comes and puts us in a corset so that we finally sit properly. […] I think it would be really cool if they did that, if you could learn how to sit properly and maybe do a few exercises with them.”

### Social interactions

Some participants wished for a reinforcement of the student body. The enhancement of mutual respect between students and teaching staff was also mentioned [I]. Finally, more guidance and support of teaching staff was proposed (e.g., being shown how to perform technical issues properly) [J], and some students wished for more qualification for teaching staff regarding their teaching competences (i.e., courses for staff on how to teach efficiently).

“I think lecturers should do a course or a training that is also related to teaching. So that they know how to teach properly.”

### Other suggestions

Additional suggestions included priorities to get course places during lecture time for students from other countries (so that they could go home and visit their families in the semester break) or considering students with child(ren) and their parental duties (e.g., giving them the opportunity to choose time preferences for courses beforehand) [K]. It was also mentioned that sports and the importance of staying fit and healthy should be promoted more [L].

## Discussion

### Summary of main findings

A broad range of stressors, resources and suggested interventions emerged from the group discussions. Many stressors were related to aspects concerning poor study organization, a perceived lack of digitalization, perceived missing reliable examination criteria, inadequate evaluation and feedback, workplaces and material and social interaction especially with (teaching) staff. Important resources encompassed family, friends and partners, appreciation, financial aid or functioning equipment. Ideas for interventions included regular exchange meetings between the different departments, improved digitalization, fixed and known evaluation criteria, options to lend material from the university or the student body, the inclusion of physiotherapy in the curriculum and the enhancement of support and teaching skills by staff.

### Comparison with prior research

Our results are consistent with previous quantitative and qualitative findings [19, 37–39], which can be taken as an indication that the observed stressors and resources occur in several dental schools in Germany and other countries. Stressors included waiting times (e.g., waiting for patients and signatures) [38], missing patients leading to the failure of a course and issues related to patient treatment [17, 40, 41], financial expenses and debts [42, 43]. Additionally, new stressors concerning, e.g., concurrent exams, obligatory brands as well as perceived tension and a lack of communication between the different departments emerged from our study. Concerning coping or relaxing activities, a previous study conducted at a different dental school in Germany found that the most favorable coping activities among dental students include contact to and exchange with friends, exercising and yoga [2]. Some students also mentioned using drugs or drinking alcohol to relax [2], which was not reported in our study, maybe due to the less confidential setting in the focus groups or social desirability. Another study from Germany assessed the resilience status of dental students and found that dental students in Germany have a lower resilience compared to medical students and that targeted resilience training is useful (e.g., modules on time and energy management, mindfulness or work-life-balance) [44].

Overall, examinations and grading procedures have been identified as a key stressor before [3, 39]. These aspects also emerged from our data. New insights were gained as students indicated that some exams did not only take place on the same day but also at the same time. Students perceived missing communication and resentments between the different departments to be the main reason for an inadequate coordination. To enhance cooperation between the departments and to avoid concurrent courses or exams, weekly or monthly exchange meetings between responsible persons of the different departments could be a useful starting point as suggested by several participants.

Evaluation, feedback and a perceived lack of anonymity as well as a fear of standing out positively or negatively were also mentioned by our participants. A previous study also found that dental students had the impression that they were perceived as impolite if they asked teaching staff for advice [45], which was also mentioned by participants in our study. Students in our study seemed to be very reluctant to give genuine feedback in course evaluations and feedback surveys and wished for more anonymity. Another study has suggested that true and anonymous feedback is helpful for dental students and that evaluative feedback is necessary to improve education and the performance of teaching staff [46]. As suggested by participants and prior research, anonymous feedback that is taken seriously should be advocated. Since participants mentioned valued feedback that led to actual changes as a resource, evaluation options could be further improved. Feedback could also be given anonymously. This is not the case, however, when evaluation sheets are to be submitted in person and/or written by hand. A previous intervention study assessed the effect of an implemented web-based evaluation system on the number of evaluations given by dental students and found a considerable increase in student feedback after the implementation of the tool [47]. Web-based evaluation tools might thus be useful in order to enhance anonymity and encourage students to give genuine feedback. Improving the overall communication between students and teaching staff to promote more courteous interactions, encouraging students to ask questions and lowering barriers to approach teaching staff might be useful as well.

Many of the observed stressors in our study were related to a perceived lack of digitalization and the impractical use of analog procedures. It can be beneficial to provide medical students with suitable digital competencies [48] as younger generations do not automatically have adequate skills to work and learn digitally [49]. A previous study documented positive attitudes towards e-learning and digital options for education and communication among dental students as early as 2013 [50]. Currently, the field of digitalization is expanding, and new options exist, e.g., novel methods of teaching dental caries removal on 3D-printed teeth [51]. Trying out and implementing new methods was also valued by participants in our study. However, it has to be kept in mind that they also wished for more support and guidance in practical teaching, especially when learning new techniques and using them for the first time.

To the best of our knowledge, there is a lack of studies on suggested and implemented interventions to reduce stress and improve well-being, learning gains and resources among dental students since most intervention studies do not cover the perspective or address the wishes of students and usually focus on individual interventions. A systematic review on mental health and well-being interventions also highlighted the general lack of research concerning possible interventions and their evaluation in the dental sector, including dental schools, as the authors found no primary prevention studies, i.e., no studies dealing with prevention that refers to changing or eliminating potential sources of stress to reduce the negative impact of stressors on individuals [13]. Studies on interventions among dental students in the review covered the introduction of a life coaching program by senior dental students, the comparison of two different psychoeducational interventions and an intervention to reduce feelings of impostorism. Additionally, two studies included dealt with counseling by a psychologist and the implementation of weekly psychotherapy sessions [13]. These suggestions were not made by our participants, which could be seen as an indication that different problems or different levels of interventions were important for them (e.g., more focus on primary prevention to eliminate sources of stress). Students in our study had a focus on both behavioral and structural prevention. Participatory interventions that are developed with students and decision-makers can be beneficial, as they are likely to take both [13] approaches to prevention into account. Minor adaptations on an organizational level might thus improve the well-being of students (e.g., anonymous feedback or agreements concerning the scheduling of exams).

There are two previous qualitative studies from our group with a similar approach that were conducted at the University of Düsseldorf with medical students and included students’ suggestions for interventions [27, 28]. In contrast to the dental study program, mostly objective examinations (e.g., multiple choice), the profound use of digital platforms, anonymous and digital evaluations and the central coordination of exams and courses have already been implemented in medical studies. Yet, medical students at the site wished for, e.g., more information on psychological counseling services, better information management with regard to contact persons as well as favored peer-to-peer mentoring programs for freshmen and self-management courses mainly related to stress and time management. Psychological counseling services were not mentioned by participants in our study, potentially due to the differences in study contents (i.e., dealing with death, suffering and emotional stress as issues that are more present among medical students). Whereas the provision of timely information and the management of information were also suggested by the participants in our study, stress and time management courses were not mentioned. For our participants, the improvement of ergonomics and prevention of potential risks of poor postures seemed to be more relevant, as they suggested the implementation of small exercises or physiotherapy sessions in their study routine or the curriculum. There are several other studies on ergonomic risk factors among dental students that also include further interventions that have not been mentioned by participants in our study: photographing dental students at work and giving them these pictures to complete self-assessments, which led to an improvement in musculoskeletal health and more awareness for posture among students [52], digital sound feedback for dental hygiene students, which proved to be effective in fostering correct working posture [53] and the analysis and prevention of ergonomic risk factors with the help of questionnaires and electromyography analyses for the activity of muscles among students during work, suggesting regular physical activity to avoid ergonomic issues [54]. Since the prevalence of musculoskeletal disorders among dentists is high [55], interventions concerning ergonomics targeting dental students might be especially useful. Implementing regular sessions to apply relaxation techniques (e.g., yoga and breathing techniques/meditation) might also provide some benefit and represent a low-threshold option to counter stress [56]. Furthermore, there is evidence to suggest that, for instance, yoga sessions are well accepted and feasible among dental and dental hygiene students [21].

Students in our study generally expressed that they perceived possible consequences of the different stressors, e.g., fear of becoming ill, a lack of free time, performance and time pressure, guilty conscience when relaxing, neglecting their health or sleep disturbances. An adequate work-life balance and sufficiently strong resources, however, are useful in order to better deal with stressful working conditions [57]. It has been found that sleeping habits of dental students can be detrimental to their academic performance. A previous study found that going to bed and getting up early as well as taking little time to fall asleep correlate with high academic performances [58]. According to the participants of our study, these conditions can often not be met since students explained that they tend to prepare for courses until late in the evening, sleep too little or suffer from sleeping disorders. Despite the perception of various stressors students also seemed to enjoy their studies, especially because of the technical aspects, the work with patients, future perspectives and positive experiences.

### Strengths and limitations

Strengths of this qualitative study include the involvement of multiple analysts in the data analysis. Additionally, participants of different semesters, different age and gender were included, which increases the likelihood that we were able to include participants with a broad range of opinions and perceptions. The proportion of men in our study was slightly lower than expected: Only nine male students took part in the focus groups. Therefore, we cannot rule out that views held by male students were explored less thoroughly than those of female students (i.e., 16% male participants). This may present a particular issue if the perceptions of stressors, resources and possible interventions differ by gender, so that further studies including more male students might be useful. Unfortunately, we were not able to recruit participants from the first semester. This might be especially beneficial for further studies, as this population might provide additional insights. Participants knew each other since they were in the same section and due to the rather small size of the study program, which could be a limitation of our study. However, this might have been a benefit as well because there was a trusting atmosphere. It can be possible that students felt uncomfortable or did not want to discuss delicate issues in front of their fellow students because anonymity amongst students was not given. Yet, despite students’ reluctance in providing genuine feedback and criticizing aspects in their studies, as they have mentioned, they willingly talked about different themes in the group discussions. This could indicate an overall pleasant group atmosphere in the discussions so that students did not fear negative consequences. This was also achieved by not addressing participants with their names but with numbers instead to ensure more anonymity regarding the audio recording. Furthermore, group discussions were not conducted in the dental faculty, and no dental staff was present to enhance confidentiality. It might also be possible that not all topics were covered, e.g., because mainly students with a lot of stress and therefore no time or little stress and larger perceived time resources participated. Group discussions might have also been more appealing to students who are communicative and who do not hesitate to speak freely and openly in front of others. Finally, we only focused on the perspectives of dental students. Adding the perspective of teaching staff could provide more in-depth insights that allow us to arrive at a more comprehensive picture of the issue. Finally, data collection took place in summer 2019. In the meantime, further changes might have occurred, potentially also due to the COVID-19 pandemic, so that additional studies seem useful.

## Implications

### Implications for dental schools

It seems that several interventions at an organizational and interpersonal level could be implemented with little effort. To enhance cooperation between the departments and to avoid concurrent courses or exams, regular exchange meetings between responsible persons of the different departments were suggested. Communication trainings could be implemented to improve the communication between teaching staff and students as well as students and patients. High-quality communication between patients and treating dentists is associated with better patient care [59]. Some suggestions related to digitalization might be challenging to implement (e.g., a complete digitalization of all patient records), whereas the utilization of existing online platforms and features (like ILIAS, a web-based learning management system often used at German universities, or the university mail) would be a low-threshold option. Course information, exam dates and learning material, for example, could be provided online for everyone and relevant details would be easily accessible.

### Implications for research

Further research that covers qualitative approaches is useful in order to detect further stressors and assess study conditions for dental students. It might be beneficial to conduct research at different sites in Germany and other countries, e.g., to examine whether some of the identified stressors in our study might be site-specific for the local dental school, also including students from the first semester who might experience different or further stressors, have other resources or suggestions for interventions. Qualitative studies among the population of dental students remain scarce, especially when it comes to suggestions for interventions or the implementation of interventions. It might also be useful to present the suggested interventions to different stakeholders and decision-makers in the university setting in order to evaluate which suggestions are deemed to be useful and feasible. Qualitative studies usually aim to capture the full range of potential views, and these in-depth analyses can draw on a limited set of participants. Follow-up studies could be quantitative with the goal of producing reliable and valid estimates (e.g., of the prevalence of stressors), which are generalizable. Such studies typically draw on larger sample sizes. Additionally, studies including the perspectives of teaching staff could be useful in order to gain broader knowledge and to explore the acceptance and feasibility of interventions among teachers.

Since the time of data collection, several adjustments have already been made at the local dental school. A study coordinator has been appointed, as suggested by several participants. Students are able to contact this person with organizational and study-related issues. Additionally, there is a new building on the clinic site where parts of the dental faculty are located now. As a result, students can work with modern technical devices and there are more workplaces with installed handpieces. This should make work for students faster and easier. Since students do not need to buy the handpiece themselves anymore, this is also a cost reduction for everyone (approximately 600€). In general, there are more places now, which might have a positive influence on the working atmosphere and the availability of course places. Additionally, the new building is near the dental clinic so that teaching staff have shorter distances to presumably allow for better supervision and support of the students. Finally, changes in teaching and the dental curriculum (e.g., integrated courses) due to the new German Medical Licensure Act (since October 2021) might also be possible, as well as improvements in digitalization or other changes since the COVID-19 pandemic. The new approbation order for dental schools in Germany might have led to further adaptations. Therefore, further studies to examine potential consequences of these changes on site and the long-term effectiveness of the interventions proposed by the students would be useful in order to gain more knowledge on how changes could affect stress among dental students in the long run.

## Conclusions

The present qualitative study makes an important contribution to the research literature concerning stressors, resources and suggested interventions in dental studies from the perspective of students affected. Suggested interventions covered an increase in the use of digital learning platforms, more communication training and improving evaluation processes. Yet, findings are limited to one single university in Germany and perspectives of teaching staff and decision-makers are not included, so that additional research is necessary. A potential follow-up study on site could continue the investigation of study conditions and gather information about possible changes due to previous adaptations (study coordinator, new German Medical Licensure Act, new facilities, possible improvements concerning digitalization also due to the pandemic). In general, more interventions are required in order to continuously improve study conditions for dental students. This can be applied to the location of our study and potentially to other dental schools in Germany and beyond, as dental students seem to face similar stressors after all.

## Supplementary Information

Supplementary Material 1.

Supplementary Material 2.

Supplementary Material 3.

Supplementary Material 4.

## Data Availability

Data cannot be shared publicly, because the transcripts may contain sensitive information. The data may be obtained from the corresponding author upon reasonable request, provided that legal frameworks are not violated and that responsibilities and confidentially have been clarified.

## Abbreviations

CN: Clara Niedworok
TM: Thomas Muth
LG: Lisa Guthardt
AL: Adrian Loerbroks
