# Supplementary material for: Stress, psychosocial resources and possible interventions: a qualitative study among dental students
Source: BMC Med Educ. 2024 Dec 18;24:1479. doi: 10.1186/s12909-024-06472-1 (PMC11653810; doi:10.1186/s12909-024-06472-1)
Supplement: Supplementary file 1 — Supplementary Material 1. [file 12909_2024_6472_MOESM1_ESM.docx]

Topic Guide – Focus Groups

1. Information in advance
2. Explaining the goal of the study and the procedure (“We need your help here”, “You are the experts”)
3. Information on data protection and confidentiality
4. Information on audio recording and anonymity (numbers instead of names)
5. Information on snacks and catering
6. Further questions?
7. Introductory questions
8. What comes to your mind when you think about stress in your studies? (Notes: Everyone needs to have a chance to answer. Make sure that the focus of the discussion does not switch to a general discussion of study conditions or the evaluation of individual courses)
9. What things were particularly troublesome for you?
10. Also, what were things that you especially enjoyed in your studies?
11. Key questions related to stress and stressors for dental students
12. What things/aspects could be improved in your studies?
13. What is especially problematic or burdensome in your studies?
14. How important is your health in relation to your studies?
15. What effect do all the requirements have on you?
16. Key questions related to resources and positive aspects of the studies
17. Who supports you or what helps you when you are having problems or a stressful time?
18. What helps you deal with all the stress?
19. Who are your contact persons?
20. What works really well, what things can you benefit from?
21. What things would you describe as fun?
22. How do you finance your studies?
23. Key questions related to possible interventions and suggestions for improvement
24. What aspects could be improved in your studies and how?
25. What suggestions do you have concerning what could be changed in dental studies based on your experiences? What could be done better?
26. What are some things that would have made or would make it easier for you to start university life?
27. Overall assessment
28. What do you think is the most important aspect (stressor, resource)?
29. What would need to be changed most urgently?
30. Reassuring: Do you feel like important aspects are still missing? Is there anything else that you would like to add?
31. Conclusion
32. Thank you for participating!
33. Outlook: Explaining what we are going to do with the data (collecting, publication, starting points for improvements…)
34. Signing informed consent
35. Handing out cinema tickets
