# Supplementary material for: Stress, psychosocial resources and possible interventions: a qualitative study among dental students
Source: BMC Med Educ. 2024 Dec 18;24:1479. doi: 10.1186/s12909-024-06472-1 (PMC11653810; doi:10.1186/s12909-024-06472-1)
Supplement: Supplementary file 2 — Supplementary Material 2. [file 12909_2024_6472_MOESM2_ESM.docx]

| **Supplementary Material 2** |
| --- |
| **Exemplary quotes concerning stressors, sorted by codes**   \| **Codes** \| **Letter** \| **Quotes** \| \| --- \| --- \| --- \| \| Organization of the study program \| A  B  C \| “Well, your problems are ignored, or they tell you: ‘Well, there’s nothing I can do there.’ And you really start to wonder, alright who’s responsible then? It feels like problems are passed on.”  “All departments decide for themselves. There is no central contact person and no central unit that organizes and coordinates things.”  “And the communication of the departments in general. There is so much bad talk about other departments in front of the students. There’s so much internal dispute. I really don’t care. They can argue as much as they want. But I think we shouldn’t suffer from it, nor should we even notice it. I don’t think that’s really professional.“ \| \| Digitalization \| D \| “All practices I know of work digitally. And for me, it doesn’t make sense, especially since this is a university clinic. We spend half the day looking for files. We carry papers from A to B and we have to ask lots of questions.” \| \| Study content/practical tasks \| E  F \| "After that, it was more learning by doing with YouTube. I think I still haven’t been sitting next to my dentist to see how to grind a tooth at first hand, without camera or TV."  "We have no psychological training, no further training, no knowledge of how to deal with people. I was given an anxious patient straight away and I had to deal with it myself somehow. We also have contact with difficult patients. Many have dental phobias or other issues. And that's completely missing. I think you're expected to get on really well with the patient, not just with the treatment, but also on a psychological level. And that doesn't make sense. […] But that's just the way it is. And that's what I’m really missing, this psychological aspect, how to deal with people." \| \| Examination system/evaluation criteria \| G  H  I  J  K \| “One of the assistants says one thing and the next one says something else […]. One of them tells you: 'Alright, you need to grind a bit more, here.” The other one says: 'No, that’s too low, you have to make a new one.’”  “This is the case, for example, when patients don't show up and are unreliable. Which holds true for most of the clinic's patients. And then, you might not pass a course or you're afraid that you won't pass the course. Because you can't find a patient who needs a crown, for example, or a root canal treatment, a total prosthesis. And because of that, you might have to extend your studies and you're in a situation of uncertainty, just hoping to find someone. And we can't influence these factors.”  “They could just take suggestions from students more seriously and really consider them. Sometimes you even get a roasting for making suggestions, or they laugh at your evaluation instead of taking it seriously.”  “I deliberately changed my writing."  "You had to hand the evaluation in together with the exam. This can easily be matched. How am I supposed to give them zero points for professional competence? You can't do that." \| \| Work/study environment \| L  M \| "Well, I think it would be easier with only five people in the course, or let’s say half of them. It's such a stressful situation. Sometimes I feel like a lab rat who doesn’t have enough space to work. We share a table with two people. This doesn’t work at all. It's just an insane level of stress."  "You pay money for each semester and a lost semester is very stressful, because of all the money you've already spent, also knowing that you won't be doing anything for a whole semester. It feels completely wasted, especially because you paid money for it." \| \| Social interactions \| N  O \| "If you're not lucky, you might end up with bad assistant doctors who can really excoriate you, because nobody cares about you, nobody is really interested in you, nobody in fact knows that you're still here."  "I think it's stupid that they don't make any effort to teach us something. Instead, they only test and rate us and that’s it." \| \| Personal stressors \| P \| "I was so sick that I fainted in the bathroom in the morning, and I woke up on the floor. Still, I dragged myself to university. Because I knew that I had to go there. I probably had a very high temperature. I guess I was contagious too. I don't know, but I had to go, even though I passed out earlier.” \| |
