# Supplementary material for: Stress, psychosocial resources and possible interventions: a qualitative study among dental students
Source: BMC Med Educ. 2024 Dec 18;24:1479. doi: 10.1186/s12909-024-06472-1 (PMC11653810; doi:10.1186/s12909-024-06472-1)
Supplement: Supplementary file 3 — Supplementary Material 3. [file 12909_2024_6472_MOESM3_ESM.docx]

| **Supplementary Material 3**  **Exemplary quotes concerning interventions, sorted by codes** |
| --- |
| \| **Codes** \| **Letter** \| **Quotes** \| \| --- \| --- \| --- \| \| Organization of the study program \| A \| “I think that there really should be someone who just takes care of solving our problems. But there's nobody there who can do that because everyone is overstrained.” \| \| Digitalization \| B  C \| "If you had everything in digital form, everyone would always know about the current status of treatment and cost plans, which of them have already been written, which have been sent out or returned. Every department could see who is treating the patient and when their next appointments are. It would facilitate so many appointments, solve so many problems."  "Some things could certainly be improved, such as simply putting things online. Lecturers from other departments in the preclinical phase have already come up with this. And then you can look at it online when you’re so busy and have your head clear." \| \| Study content/practical tasks \| D  E \| “I've heard from other universities that some preparation rooms are opened up again in the evening, so that those who feel the need can simply come back in the evening to practice more.”  “Okay, you could make a short movie [...]. We also saw that once in another place. [...] That was just a really short broadcast, a simple report where students were interviewed, and it was explained that patients can be treated in the student course and what kind of services there are.” \| \| Examination system/evaluation criteria \| F  G \| "Just tell me whether I passed or failed the course. And if I didn't pass it, I would like to have a private discussion where they tell me what wasn't good."  "They should just sit down together, the assistants and the professor, for example, and talk about it: "Let’s see. We want to have this amount of erosion from the tooth. It should have this shape" and so on. Just defining it to prevent everyone from saying something else." \| \| Work/study environment \| H \| "Other universities also offer the option of borrowing materials, for example. That you pay a loan fee, for a phantom head or for a handpiece, for example. I think this would be a good offer for the students. You pay some kind of loan fee and receive an instrument that is intact. You give it back and you get the loan fee back. I think you could offer something like that somehow instead of buying everything yourself." \| \| Social interactions \| I  J \| “If you address the other person formally, they should do the same. It's weird when it's not on the same level, I think, especially if the person is as old as you are. It's just awkward to be addressed informally then.”  “It would be much nicer if there were some kinds of additional instructions. There are demonstrations once a week. But they don’t always take place and they’re just for half an hour. So, I think there should be more teaching. And less testing and evaluating.” \| \| Other suggestions \| K  L \| "The foreign students who have to fly home and of course want to spend their free time with their families in the semester break [...] That, if possible, they are prioritized and can take courses in the semester."  "It might be an idea for the university to somehow give us the right mindset from the beginning onwards, to promote sports, take care of yourselves." \| |
