# Supplementary material for: Stress, psychosocial resources and possible interventions: a qualitative study among dental students
Source: BMC Med Educ. 2024 Dec 18;24:1479. doi: 10.1186/s12909-024-06472-1 (PMC11653810; doi:10.1186/s12909-024-06472-1)
Supplement: Supplementary file 4 — Supplementary Material 4. [file 12909_2024_6472_MOESM4_ESM.docx]

**Resources**

*Organization of the study program and digitalization*

Participants perceived good course organization and adequate supervision as helpful. They also appreciated having less workload and waiting times.

"These small feelings of success that you have are nice. Sometimes you get a signature you need straight away. Or you're happy, because there's no queue and you can instantly talk to the assistant."

Participants perceived it as positive when some departments tried implementing modern techniques. In general, material and technological features for dental care were seen as suitable and beneficial for patients provided, they were used.

“You can see that they are trying to introduce new things, new methods and techniques. And high-tech scanners, 3D printing, these sort of things. We respect that and you can see that they are trying.”

*Study content/practical tasks*

Participants appreciated making their own decisions based on prior experiences (e.g., in treating patients). This seemed to enhance confidence and contentment.

"As soon as you have a certain amount of experience, and I also liked that in some courses, they let you do things. If you are a student who has already done two or three clinical courses, you can do it. And I think that’s good."

Additionally, students positively talked about qualifications and experiences in the study program.

"I think it's good that we get a lot of practical experience in our studies, so that we can gain a lot of experience and are not on our own in the practices after the studies and don’t have to do everything for the first time then. So, we are supported here to a certain extent, we can attend accompanying lectures and seminars and we also have the opportunity to ask questions."

They especially liked practical courses, working with patients and personal progress, which seemed to give them a positive feeling and prepare them for their later work routine (i.e., not scared of eventually being overwhelmed as dentists due to adequate training and a broad fundamental knowledge).

"Someone has already mentioned that you're getting better and better on the course compared to the first semester. And seeing that is just so much fun."

*Examination system/evaluation criteria*

Students perceived it as helpful to have enough time for tasks and submissions, which seemed to lead to less stress. They also greatly appreciated feedback that was taken seriously and led to actual changes. They explained that they felt valued this way and had the impression of having an impact on their study.

"It's really positive for us, we write an evaluation, and you can tell the difference. We had one that was really negative. And now you can see that they got other assistants. So, it's useful, the students' opinions are really valued in this case, and you can see that it's useful."

*Work/Study environment*

Participants appreciated an organized and clean work environment, functioning workplaces and the availability of material (e.g., working burners, good microscopes). They also positively emphasized modern or new buildings (e.g., the new pre-clinic being built, the canteen or the library with a good selection of literature).

"Well, the old pre-clinic will soon be gone, thank God. I think that will solve half of the problems anyway. We’ll have more space, more preparation places, we’ll have functioning workplaces."

"And the things I found positive were, for example, the great library we had and all the books we could borrow. That was an advantage for me. I was really looking forward to it. There's some really nice literature and you can learn really well."

*Social interactions*

Participants talked about individual positive experiences with patients (e.g., great feeling of helping others, polite patients) and teaching staff (e.g., support, guidance, positive feedback). They positively perceived staff, to whom they could come with questions without fear of negative feedback, who could teach them something and in whom they could confide.

"There really are a lot of genuine people here. And you can learn something from them. You can ask questions to them. You can also somehow confide in them."

Students also valued staff who seemed to take them and their problems seriously, who were friendly and polite.

"One professor, for example, wants to have issues with the plans on medical expenses clarified and resolved faster. And for the first time in a month and a half I feel like someone is really interested in problems we have."

Fellow students, the student body and the semester speaker were also valued by the participants. Some mentioned great mutual support and friendship among students (assistance in practical courses, having someone to talk to).

"We consider us as a team, and I am happy to sacrifice my study time so that we can help each other."

Participants felt that they could rely on each other and seemed to gain confidence and assurance when talking to fellow students (e.g., confirmation, approval). This was perceived as energetic and seemed to give students strength.

"I really like that we are a community, and we all have the same problems, which is not necessarily positive, but you have people who are in the same situation as you. And you have that sense of community somehow, as you know we're all in the same situation. We all want to get through it together. And I think friends are a great help, also family, of course. And I think that really gives you strength too."

*Individual and other resources*

Students positively talked about enjoying studying and being motivated by prospects of becoming a dentist. Social aspects and resources related to their free time were also perceived as helpful (e.g., family, partner and friends, doing sports, travelling). Furthermore, some mentioned a strong will and the ambition of not wanting to give up as motivating.

“Eventually, you’ll reach the point where you think: Alright, I bought all the suitcases second-hand, but I paid so much money for them. It’s so much money for each semester, a lot of material costs. I could have worked in that time. I won’t give up now, because I’ve come so far. And everything would have been in vain. That's my motto."

Finally, participants explained that financial resources also seemed to be an essential component to successfully manage the studies (i.e., earning money in a part-time-job, receiving money from their parents, getting student loans or funding under the Federal Training Assistance Act (German BAföG)).

"I would say that many of us are luckily in the position of being a daughter or a son and, thank God, we get financial support by our parents."
